# Measurement of gamma radiation levels in soil samples from Thanjavur using γ-ray spectrometry and estimation of population exposure

**DOI:** 10.4103/0971-6203.55966

**Published:** 2010

**Authors:** B. Senthilkumar, V. Dhavamani, S. Ramkumar, P. Philominathan

**Affiliations:** 1 Institute for Ocean Management, Anna University Chennai, Chennai - 600 025, India; 2 Department of Physics, AVVM Sri Pushpam College, Poondi, Thanjavur - 613 502, India; 3 Environmental Survey Laboratory, Health Physics Division, MAPS, Kalpakkam - 603 102, India

**Keywords:** Absorbed dose, external hazard index (Hex), gamma radiation, natural radioactivity, soil, γ-ray spectrometry

## Abstract

This study assesses the level of terrestrial gamma radiation and associated dose rates from the naturally occurring radionuclides ^232^Th, ^238^U and ^40^K in 10 soil samples collected from Thanjavur (Tamil Nadu, India) using γ-ray spectrometry. The activity profile of radionuclides has clearly showed the existence of low level activity in Thanjavur. The geometric mean activity concentrations of ^232^Th, ^238^U and ^40^K is 42.9±9.4 Bq.kg^−1^, 14.7±1.7 Bq.kg^−1^ and 149.5±3.1 Bq.kg^−1^ respectively are derived from all the soil samples studied. The activity concentration of ^232^Th, ^238^U and ^40^K in soil is due to the presence of metamorphic rocks like shale, hornblende-biotite gneiss and quartzofeldspathic gneiss in these areas. Gamma absorbed dose rates in air outdoors were calculated to be in the range between 32 nGy.h^−1^ and 59.1 nGy.h^−1^ with an arithmetic mean of 43.3 ±9 nGy.h^−1^. This value is lesser than the population weighted world-averaged of 60 nGy.h^−1^. Inhabitants of Thanjavur are subjected to external gamma radiation exposure (effective dose) ranging between 39.2 and 72.6 *μ*Sv.y^−1^ with an arithmetic mean of 53.1±11 *μ*Sv.y^−1^. The values of the external hazard index determined from the soil radioactivity of the study area are less than the recommended safe levels.

## Introduction

The natural terrestrial gamma radiation dose rate is an important contribution to the average dose rate received by the world's population.[1,2] Estimation of the radiation dose distribution is important in assessing the health risk to a population and serve as the reference in documenting changes to environmental radioactivity in soil due to anthropogenic activities.[3] Human beings are exposed outdoors to the natural terrestrial radiation that originates predominantly from the upper 30 cm of the soil.[4] Only radionuclides with half-lives comparable with the age of the earth or their corresponding decay products existing in terrestrial material such as ^232^Th, ^238^U and ^40^K are of great interest. Since these radionuclides are not uniformly distributed, the knowledge of their distribution in soil and rock play an important role in radiation protection and measurement.[5] Gamma radiation from these represents the main external source of irradiation to the human body and the concentrations of these radionuclides in soil are determined by the radioactivity of the rock and also nature of the process of the formation of the soils.[6,7] Therefore, radionuclides in soil generate a significant component of the background radiation exposure to the population.[8]

The aim of this work is to measure the specific activity and γ- ray absorbed doses of the naturally occurring radionuclides (^238^U, ^232^Th and ^40^K) in different types of soils from Thanjavur (Tamil Nadu) using γ- ray spectrometry. This was accomplished through the following types of measurements: radionuclide activity concentrations in surface soil, outdoor gamma absorbed doses and the external hazard index (H_ex_) for Thanjavur.

## Materials and Methods

### Physiographic Setting

**Figure 1:**
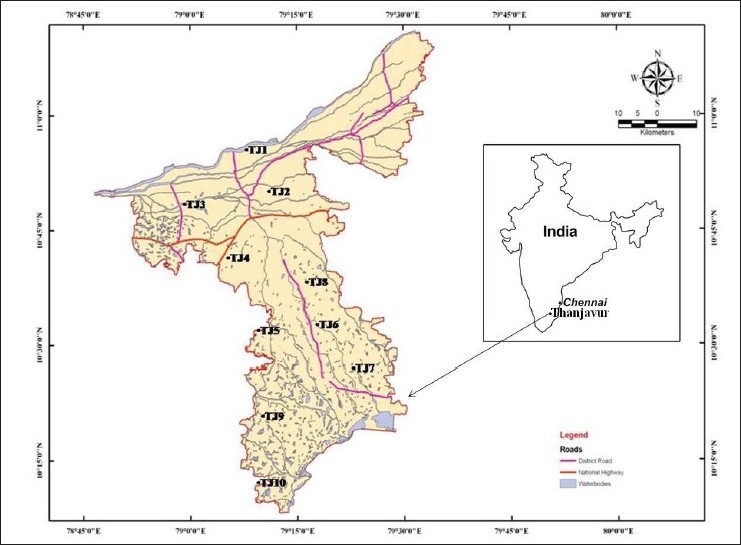
Map Showing Sampling Locations in Thanjavur (Tamil Nadu)

Thanjavur is located at 10°47' N and 79°10'E [Figure 1]. The city lies on the Cauvery river basin around 200 miles south of Tamil Nadu state capital Chennai. The city spreads in an area of 36.3 km^2^. As per the latest census 2001, the population of Thanjavur reached 0.2 million which is contributing 40% to the total population of the Thanjavur district. The density of the city population is 5508 persons per km^2^. The geological formation of the Thanjavur is made up of cretaceous, tertiary and alluvial deposits and the major area are occupied by the alluvial and tertiary deposits. The cretaceous formations occur as a small patch west and south-west of Vallam. These formations have a very thick lateritic cap, consisting of impure limestones and sand stones of silt, clay calcareous and argillaceous variety. In the coast these formations are overlain by Cuddalore sand stones of tertiary age. These sand stones are covered by a thin layer of windblown sandy clay, unconsolidated sand; clay bound sand and mottled clay with lignite seams. This tertiary formation is invariably capped by laterite. In the east, the alluvial deposits of the river Cauvery and its tributaries lie over the tertiary sand stone. They consist of medium to fine sand, gravelly sand, clay and sandy clay. The thickness of these formations ranges from 30 m to 400 m.

### Sample Collection and Preparation Techniques

Ten sampling locations were chosen from all over the city to conduct the radiometric study [Figure 1]. Out of these, samples of black soil were collected from three locations, red soil from four and alluvial loam soil from the remaining three. The bulk soil samples were collected in undisturbed, uncultivated, grass covered level areas and in remote locations from man-made structures such as roads and buildings to prevent any external influence on the results. Each soil sample was collected from nine subsamples in an area of approximately 100 m^2^ and up to a depth of 10 to 15 cm. The subsamples were mixed thoroughly and were collected in polythene bags. The homogenized soil samples were then oven dried at 60 - 80°C for about 24 hours. The dried samples were ground with mortar and pestle and then allowed to pass through a 100-mesh sieve. In order to maintain radioactive equilibrium between ^226^Ra and its daughters, the soil samples were then packed in a 250 ml air tight PVC container, dry-weighed and stored for a period of one month for equilibrium. Each sample was then counted using a gamma spectroscopy device.

### Gamma-ray Detection System

The gamma spectrometric measurement was carried out using HPGe Gamma ray spectrometric system at the Environmental Survey Laboratory, Health Physics Division, BARC, Kalpakkam, India. The detector is mounted vertically coupled with 8K PC based multi channel analyzer (MCA) and the detector is enclosed in a massive lead shield to reduce background of the system. IAEA standard reference materials, Uranium ore (RGU-1) Thorium ore (RG Th-1) and KCl powder of known activity, were used for calibration of the system. The spectrometer was calibrated for energy and efficiency over energy range 200keV to 3MeV. Each sample was counted for 20,000 seconds to reduce the statistical uncertainty. Minimum measurable activity was determined from the background radiation spectrum and it is 1 Bq.kg^−1^ for ^238^U, 3 Bq.kg^−1^ for ^232^Th and 38 Bq.kg^−1^ for ^40^K with 99% of confidence interval. The peak corresponding to 1.46 MeV for ^40^K, 1.76 MeV (^214^Bi) for ^238^U series and 2.61 MeV (^208^Ti) for ^232^Th are considered for the estimation of natural radionuclides. The activity of each sample was determined using the total net counts under the selected photopeaks after subtracting appropriate background counts and applying appropriate factors for photopeak efficiency, gamma intensity of the radionuclide and weight of the sample.[9,10] The gamma absorbed dose in air at a height of one meter above ground surface is estimated from the activity concentrations of gamma emitting isotopes present in the soil.

## Results and Discussion

### Radioactivity Concentration Levels

The radionuclide composition for some of the collected soil samples indicates the variability of geological formations for the area studied. Table 1 illustrates the specific activity of the natural radionuclides (^238^U, ^232^Th, and ^40^K) in the samples and Table 2 gives the statistics of values corresponding to specific activities measured for the ^232^Th series, ^238^U series and ^40^K in the surface soil samples collected at different parts of Thanjavur. The specific activity of radionuclides in soil is given in Bq kg^−1^ dry weight. ^232^Th activity in the soil samples is distinctly higher than that of ^238^U and it ranges between 18.6 Bq.kg^−1^ and 76.6 Bq.kg^−1^ with a geometric mean activity of 42.7±9.4 Bq.kg^−1^. ^238^U concentration in the soil samples ranges between 7.3 Bqkg^−1^ and 24.7 Bqkg^−1^ with a geometric mean activity of 14.7±1.7 Bq.kg^−1^ and found to be lesser than that of both ^232^Th and ^40^K. The activity of ^40^K is observed comparatively higher than that of both ^232^Th and ^238^U in all sampling locations studied and it is ranges between 38 Bqkg^−1^ and 417 Bqkg^−1^ with a geometric mean activity of 149.5±3.1 Bqkg^−1^. In the present study the distribution of radionuclides in soil samples are asymmetrical [Table 2].

**Table 1:** Mean specific activities of ^232^Th ^238^U and ^40^K for Thanjavur

*Sample code*	*Location Name*	*Soil type*	*^232^Th (Bq kg^−1^)*	*^238^U (Bq kg^−1^)*	*^40^K (Bq kg^−1^)*
TJ1	Manambu Chavadi	Alluvial	44.7±5.4	16±0.8	219 ±6.3
TJ2	Thanjavur Jn.	Red soil	46.7±3.4	19.1±1.3	89±0.7
TJ3	Pambatti Street	Alluvial	30±7.7	10.1±0.9	234±0.7
TJ4	Arulananda Nagar	Red Soil	48.6±9.9	24.6±2.0	38±5.2
TJ5	Tamil University	Red soil	45.5±12.4	24.7±6.2	46±3.2
TJ6	Medical College	Red Soil	69.7±7.4	21.6±1.3	133±2.6
TJ7	Srinivasapuram	Black soil	76.6±13.5	11.5±1.7	181 ±8.0
TJ8	South Rampart	Black soil	40.7±14.1	15.1±2.0	236±2.1
TJ9	Rajagori	Black soil	18.6±13.9	7.3±3.1	417±7.5
TJ10	Karanthai	Alluvial	36.2±16.1	9.3±2.1	290±6.5

**Table 2:** Statistical data for radioactivity concentrations of ^232^Th Series, ^238^U series and ^40^K in surface soil samples from Thanjavur

	*Activity concentration (Bq.kg^−1^ dry wt)*		
	
	*^232^Th series*	*^238^U series*	*^40^K*
Average	45.7	15.9	188.6
Range	18.6 - 76.6	7.3 - 24.7	38 - 417
Skewness	0.5	0.2	0.5
Kurtosis	0.3	-1.4	0.1
Frequency distribution	Normal	Normal	Normal

The results obtained in this study are comparable to worldwide average concentration of these radionuclides in soils reported by UNSCEAR,[11] which are 40 Bq kg^−1^ for ^238^U and ^232^Th and 370 Bq kg^−1^ for ^40^K. The highest concentration of ^232^Th was observed in sampling areas TJ6 and TJ7 may be due to the presence of metamorphic rocks like shale, Hornblende-biotite gneiss and Quartzofeldspathic gneiss in these areas. However, a detailed geochemical investigation is required to reach at some conclusion. The abundance of ^40^K activity was observed in predominantly agricultural areas in the outskirts of the city due to the use of potassium fertilizers and also in the remaining areas because of geological origin. In addition to this, ^238^U and ^232^Th concentration was found to be high in red soil whereas, ^40^K found to high in alluvial soil. The concentration activity of ^232^Th, ^238^U and ^40^K measured in Thanjavur is compared with that of other cities within and outside India are presented in Table 3.

**Table 3:** Comparison of Activity Concentration of ^232^Th, ^238^U and ^40^K in soil samples of Thanjavur and other parts of India

*Location*	*Activity concentration (Bq/kg)*			*Reference*
		
	*^232^Th*	*^238^U*	*^40^K*	
Thanjavur, South India	18.6 - 76.6 (45.7)	7.3 - 24.7 (15.9)	38 - 417 (189)	Present study
Kalpakkam, South India	15 - 776 (119)	5 - 71 (16)	200 - 854 (406)	Kannan *et al*.[17]
Kudankulum, South India	334.2	-	348.49	Brahmanandhan *et al*.[18]
Gudalore, South India	19 - 272	17 - 62	78 - 596	Selvasekara Pandian *et al*.[20]
Himachal Pradesh, India	52.8-105.81 (82.2)	-	95.3 - 160.3 (135.7)	Rani and Singh[5]
India Average	18.3	14.8	–	Mishra and Sadasivaxm[10]
World Range	7 - 50 (25)	10 - 50 (25)	100 - 700 (370)	UNSCEAR[19]

### Gamma Absorbed Dose Rates

A relevant quantity when considering radiation risk to humans and other biota is the absorbed dose rate.[12] The absorbed dose rate, D (nGy h^−1^), at a height of 1 m above the ground surface due to the concentrations of ^238^U, ^232^Th and ^40^K in the soil in all sampling locations is presented in Table 4. The dose can be calculated using the absorbed dose rate activity conversion factors depending on the radionuclides studied in the soil. The conversion factor described by UNSCEAR[11] was adopted and the gamma absorbed dose rates calculated using the equation^12^ given below:

**Table 4:** Air-absorbed Dose Rates and Annual Effective Doses at Various Locations of Thanjavur

*Sample code*	*Absorbed dose rate (nGy.h^−1^)*	*Annual effective dose (μSv.y^−1^)*	*External hazard index (H_ex_)*
TJ1	43.53	53.42	0.26
TJ2	40.74	50.00	0.25
TJ3	32.58	39.98	0.19
TJ4	42.30	51.91	0.26
TJ5	40.83	50.10	0.25
TJ6	57.64	70.73	0.36
TJ7	59.16	72.59	0.36
TJ8	41.40	50.81	0.25
TJ9	32.00	39.26	0.18
TJ10	38.29	46.99	0.23
Range	32.00 - 59.16	39.27 - 72.60	0.18 - 0.36
Average	43.30	53.14	0.26

(1) $$\text{D}=\left(0.604{\text{ C}}_{\text{Th}}+0.462{\text{ C}}_{\text{U}}+0.0417{\text{C}}_{\text{K}}\right).\text{ nGy}.{\text{h}}^{-1}$$

Where C_Th_, C_U_ and C_K_ are the activity concentrations of primordial radionuclides viz., ^232^Th, ^238^U and ^40^K existing in the soil in Bq.Kg^−1^.

**Figure 2:**
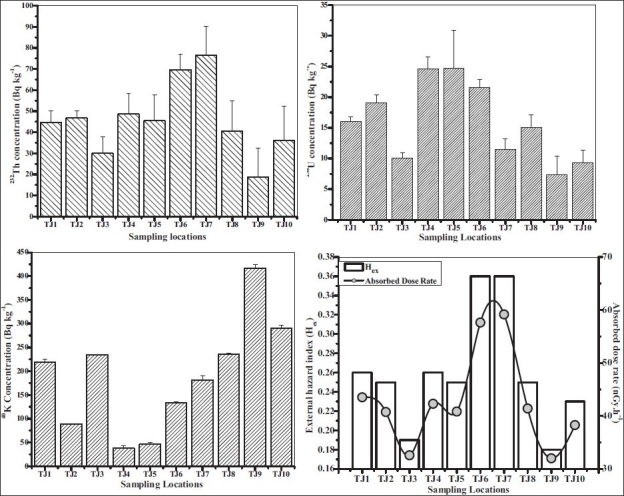
Activity concentration of measured primordial radionuclides, absorbed dose rate and external hazard index for Thanjavur

From equation (1), the dose contribution per unit activity concentration of ^232^Th to ^238^U to ^40^K is in the ratio of 1:0.6:0.06. The average outdoor gamma absorbed doses in air calculated from the concentrations of each of the nuclides of ^232^Th and ^238^U series, and of ^40^K. The outdoor gamma absorbed doses in air ranging between 32 nGy.h^−1^ and 59.1 nGy.h^−1^ with an average of 43.3± 9 nGy.h^−1^ for Thanjavur was observed, which is less than the world average value of 60 nGy.h-^111^ [Figure 2]. The differences are considered to be due to the geological settings and land use patterns, which vary from one place to another and from one locality to another in the same zone. The mean dose rate is important for determining radiation detriment to the population as a whole, but some members of the population may receive higher doses due to high concentration of radionuclides. A common feature in any environmental radiation measurements is the considerable variation in soil radioactivity with location depending on soil physiochemical parameters. Therefore the largest contribution from natural radionuclides in Thanjavur soil samples to the absorbed doses in air is due to ^232^Th.

Finally, to make a rough estimate for the annual ambient dose, one has to take into account the conversion coefficient from absorbed dose in air to effective dose and the outdoor occupancy factor. In UNSCEAR reports[2,11,14] the committee used 0.7 Sv.Gy^−1^ as the conversion coefficient from absorbed dose in air to effective dose received by adults, and 0.2 for the outdoor occupancy factor. Effective dose rate (*μ*Sv.y^−1^) due to natural activity in the soil was calculated by:

(2) $$\text{Effectivedose}=\text{Dose rate}\left(\text{nGy}{.\text{h}}^{-1}\right)\times 24\left(\text{h}\right)\times 365.25\left(\text{d}\right)\times \text{rate}\left(\mu \text{Sv}{.\text{y}}^{-1}\right)$$

0.2 (occupancy factor) × 0.7Sv.Gy^−1^ (conversion coefficient) × 10^−3^

In estimating the effective dose in any environment, the two factors of importance are the conversion coefficient from Gy h^−1^ to Sv h^-1^ and the occupancy factor. The former gives the equivalent human dose in Sv y^−1^ from the absorbed dose rate in air (Gy h^−1^) while the latter gives the fraction of the time an individual is exposed to outdoor radiation. The first factor has been recommended by the UNSCEAR[11] as 0.7 Sv Gy^−1^ and the second factor as 0.2, which suggests that from absorbed dose in air to effective dose received by adults and considering that people in India, on the average, spent ∼20% of their time outdoors, the annual effective doses are calculated. This factor suits the pattern of life in the studied area, yielding the outdoor effective dose given in table 4. Indoor dose rates were not evaluated because the essential data on average buildup of radon gas in the indoor atmosphere were not available. The corresponding outdoor annual effective doses range from 39.2 to 72.6 *μ*Sv.y^−1^ with an average value of 53.1 ±11*μ*Sv.y^−1^ were calculated for Thanjavur, while the worldwide average annual effective dose is approximately 0.5 mSv.y^−111^ and the results for individual countries being generally within the 0.3-0.6 mSv range. Thus, our results are one order magnitude less (0.05 mSv.y^−1^) than the average worldwide limits as reported by UNSCEAR.[11]

### Estimation of External Hazard Indices Due to ^238^U and ^232^Th

Many radionuclides occur naturally in terrestrial soils and rocks and upon decay, these radionuclides produce an external radiation field to which all human beings are exposed. In terms of dose, the principal primordial radionuclides are ^232^Th, ^238^U and^40^K. Both ^232^Th and ^238^U head series of radionuclides that produce significant human exposures. The decay of naturally occurring radionuclides in soil produces a gamma-beta radiation field in soil that also crosses the soil-air interface to produce exposures to humans. The main factors which determine the exposure rate to a particular individual are the concentrations of radionuclides in the soil, the time spent outdoors. In this study, the external hazard index H_ex_, is calculated and examined according to the following criterion:[15]

(3) $${\text{H}}_{\text{ex}}={\text{C}}_{\text{U}}/370+{\text{C}}_{\text{Th}}/259+{\text{C}}_{\text{K}}/4810\leq 1$$

The value of H_ex_ must be lower than unity to keep the radiation hazard insignificant. The calculated values of H_ex_ for the soil samples studied ranged between 0.18 and 0.36 with an average value of 0.26± 0.06 [Table 4, Figure 2]. These values are far below the criterion limit (H_ex_ less than or equal to one) as per the European Commission on Radiation Protection[16] reports, the terrestrial soils from this city has no high exposure for either inhabitants and can be used as a construction material without posing any significant radiological threat to the population.

## Conclusion

Gamma ray spectrometry has been used to determine the soil radioactivity concentrations of ^232^Th, ^238^U and ^40^K in 10 soil samples collected from Thanjavur, Tamil Nadu. The activity profile of radionuclides has clearly showed the existence of low level activity in Thanjavur. The mean activity concentrations of ^232^Th, ^238^U and ^40^K is 45.7±17.1 Bq.kg^−1^, 15.9±6.3 Bq.kg^−1^ and 189±117.3 Bq.kg^−1^ respectively, are derived from all the soil samples studied. These values fall within the lowest range of those measured at worldwide scale reported by other authors.

Gamma absorbed dose rates in air outdoors were calculated to be in the range 32 nGy.h-^1^ to 59.1 nGy.h^−1^ with an overall mean value of 43.3± nine nGy.h^−1^, this value is lesser than the population weighted world-averaged of 60 nGy.h^−1^. Inhabitants of the studied area are subjected to an external gamma radiation exposure (effective dose) which ranges from 39.2 to 72.6 *μ*Sv.y^−1^ with an arithmetic mean value of 53.1±11 *μ*Sv.y^−1^.

The values of the external hazard index determined in the soil of the study area are less than the recommended safe levels. Therefore, the terrestrial soils from Thanjavur have no high exposure for either inhabitants and can be used as a construction material without posing any significant radiological threat to the population. This work has been able to establish baseline information on the natural radionuclides concentrations in Thanjavur, which will serve as a reference for future assessment. Further study may be necessary to estimate internal doses and external doses from other sources for the population of Thanjavur.
